# Synthesis, characterization and potential sensing application of carbon dots synthesized via the hydrothermal treatment of cow milk

**DOI:** 10.1038/s41598-022-26906-4

**Published:** 2022-12-28

**Authors:** Avinash Kumar, Ishant Kumar, Arvind K. Gathania

**Affiliations:** Department of Physics and Photonics Science, National Institute of Technology Hamirpur, Hamirpur, HP 177005 India

**Keywords:** Nanoscience and technology, Physics

## Abstract

Carbon quantum dots (CQDs) were synthesized in this study by hydrothermally treating cow milk. The procedure is simple, non-hazardous to the environment, and does not necessitate the use of any special instruments or chemicals. CQDs were practically almost circular when they were manufactured and had an average size of 7 nm. Carbon (67.36%), oxygen (22.73%), and nitrogen (9.91%) comprised the majority of their composition. They feature broad excitation-emission spectra, excitation-dependent emission, and temperature-dependent photoluminescence. They remained quite stable in the presence of a lot of salt, UV radiation, and storage time. Because luminescence quenching mechanisms are sensitive to and selective for Sn^2+^, they can be employed to create a nanosensor for detecting Sn^2+^.

## Introduction

Carbon quantum dots (CQDs) are carbon nanoparticles smaller than 10 nm in size. They have amorphous to nanocrystalline cores and are typically quasi-spherical in shape. They are made of graphene and graphene oxide sheets using *sp*^3^ hybridized carbon insertion or *sp*^2^ graphitic carbon insertion^1–5^. Prior to CQDs, conventional dyes and semiconductor quantum dots were in use. However, their clinical applications are limited because of the utilization of highly hazardous heavy metal ions in their manufacturing^1,6–10^. This leads to a thorough analysis of CQDs. CQDs feature fluorescence qualities similar to semiconductor quantum dots, as well as minimal toxicity, low production cost, biocompatibility, and chemical inertness. They were discovered by chance in 2004 during the electrophoresis purification of single-walled carbon nanotubes, and by laser ablation of cement and graphitic powder in 2006^11^. They have recently received a lot of attention because of their unique properties like low toxicity, biocompatibility, tunable fluorescence, water-solubility, flexible surface modification^12^, and a wide range of applications like chemical sensing, bioimaging, biosensing, nanomedicine, photocatalysis, drug delivery, fluorescent probes, and optoelectronic devices^13^.

To date, several approaches for the synthesis of CQDs are available. There are two techniques for CQD synthesis in general: top-down and bottom-up. Bulk material is broken down to form nano-sized particles using the top-down technique. The Bottom-up technique, on the other hand, entails the formation of nano-sized particles by assembling atoms or molecules into useful shapes. Arc discharge^14^, Laser ablation^15,16^, Carbonization, Solvothermal^17^, Hydrothermal^18–24^, Microwave^25^, Ultrasonication method^26^, Pyrolysis^27^, Electrochemical method^28^ and chemical oxidation are among these approaches. The Hydrothermal method has recently received a lot of attention because of its low cost, biocompatibility, high efficiency, and environmental friendliness. A precursor is delivered to an autoclave reactor and allowed to react at high temperatures and pressures in a hydrothermal process. To date, CQDs were synthesized using a variety of resources such as agricultural waste, organic compounds, hazardous chemicals, natural goods, and so on. Natural precursors have received widespread interest in these fields because they are readily available, cost-effective, and environmentally acceptable. Orange juice^29^, Sugarcane juice^30^, Apple juice^31^, Lemon juice^32,33^, Coffee grounds^34^, Sweet pepper^35^, Bamboo leaves^36^, Hair^37^, Konjac flour, Grass^38^, Egg, Soya milk^39^, Cocoon silk, Garlic^40^, Red lentils^12^, and Glucose have all been used to make CQDs. Mehta et al.^41^ synthesized CQDs from apple juice for imaging mycobacterium and fungal cells. Hoan et al.^42^ prepared CQDs from lemon juice and used them as a probe for Mo^6+^ ion detection. CQDs were synthesized from red lentils by Zubair et al.^12^ for Fe^3+^ sensing. Thambiraj et al.^43^ prepared CQDs from sugarcane bagasse pulp using the chemical oxidation and exfoliation method. Rui-Jun et al.^44^ created CQDs from polyethylene glycol for use in cellular imaging. Aye Myint et al.^45^ used the ClCO_2_ antisolvent technique to create CQDs from kraft lignin, followed by carbonization and chemical oxidation for HeLa cell imaging. Hong et al.^46^ synthesized nitrogen-doped carbon nanoparticles from strawberry juice to detect mercury ions. Sen Lui et al.^47^ synthesized CQDs from grass hydrothermal treatment as a fluorescent sensing platform for label-free detection of Cu^2+^ ions. Betha et al.^48^ made fluorescent carbon dots from Carica papaya juice to image Bacterial and Fungal cells. Yang et al.^49^ created lignin-based CQDs for thermal energy storage applications.

Sn^2+^ has been utilized in dentistry to prevent tooth cavities since the 1950s^50,51^. Because of its importance to humans, its biological roles have recently been explored. It can be found in the human brain, liver, and spleen^52–54^ and is involved in growth or cancer prevention^50^. Its overabundance can have an adverse effect on the digestive and respiratory systems, while a deficit can result in hearing loss or dysplasia^54^. As a result, detecting Sn^2+^ with high sensitivity and selectivity is critical. Traditional techniques for Sn^2+^ sensing including high-performance liquid chromatography (HPLC)^55^, colorimetric detection^56^, and anodic stripping voltammetry (AVS)^57^ have drawbacks like the requirement of sophisticated instruments and a long detection time. Fluorescence sensors based on fluorescence quenching methods have been found to be a good substitute for Sn^2+^ detection due to their ease of use, high sensitivity, and quick responses^58,59^.

We used a hydrothermal approach to synthesize CQDs from cow milk. CQDs have a quasi-spherical shape with an average size of 7 nm. They demonstrated excitation-dependent emission, temperature-dependent photoluminescence (Pl), and excellent photostability. They were also looked into for metal ion sensing applications. Their sensitivity to various metal ions was studied, and they were discovered to be sensitive to Sn^2+^. As a result, they can be used to build a nanoprobe sensor for detecting Sn^2+^.

## Materials and methods

### Ethical considerations

An ethical committee’s approval was not required for the use of cow's milk in this study because the research was conducted not on animals but on their milk, which we got from the cow's owner.

### Materials

In the Supplementary File, Image 1 depicts the cow from whom the milk was obtained. It is of the Jersey breed. It belongs to Mr. Rakesh Kumar, a resident of the Hamirpur district of Himachal Pradesh, India. The milk was taken directly from the cow by milking it with the help of Mr. Rakesh. Deionized water, Sodium hydroxide (NaOH, > 96%), Sodium sulfite (Na_2_SO_3_, > 98%), hydrogen peroxide (H_2_O_2_, 30%), Anhydrous ethanol, Metal salts SnCl_2_·2H_2_O (98%), CdCl_2_·H_2_O (99%), ZnCl_2_ (98%), KCl (99%), CaCl_2_·2H_2_O (98%), NaCl (99%), FeCl_3_ (98%), LiCl (99%), and HgCl_2_ (98%) were purchased from Sigma-Aldrich. All of the chemicals were of analytical grade and did not require further purification.

### Preparation of CQDs

Stir vigorously for 20 min after adding 15 mL of deionized water to 19 mL of cow milk. Transfer this solution to a 50 ml Teflon-lined autoclave reactor and place it in a muffle furnace at 180 degrees for two hours. Allow the reactor to cool to room temperature naturally. After the carbonization of the precursor, the obtained solution was centrifuged at 8000 rpm for 30 min and the supernatant is further filtered with a 25 mm/0.2 µm syringe filter.

### Sn^2+^ detection process

After mixing 100 µL CQDs and 500 µL sodium-acetate buffer solution (pH = 7), different concentrations of metal ions and Sn^2+^ were added. With deionized water, the final volume was increased to 2.5 mL. Pl spectra were collected after 5 min of incubation.

### Characterizations

HR-TEM study was carried out by using the FEI company of USA (Model: FP 5022/22-Tecnai G2 20 S-TWIN) instrument. XPS analysis was performed on Thermofisher scientific (Model: Nexsa base) with Al K_α_ X-rays. FTIR spectra were taken on a Perkin-Elmer spectrum 65 spectrometers.PL studies were performed on Shimadzu RF-6000 Spectro fluorophotometer instrument equipped with a Xenon lamp. Photostability tests were performed with a light of 365 nm from an ultraviolet lamp at room temperature. UV–visible spectra were acquired with Vis–NIR spectrophotometer (Make: PerkinElmer Model: UV-2450).

### Owner’s consent statement

The owner of the cow gave us permission to use its milk in an experiment.

## Results and discussions

### HR-TEM study

**Figure 1:**
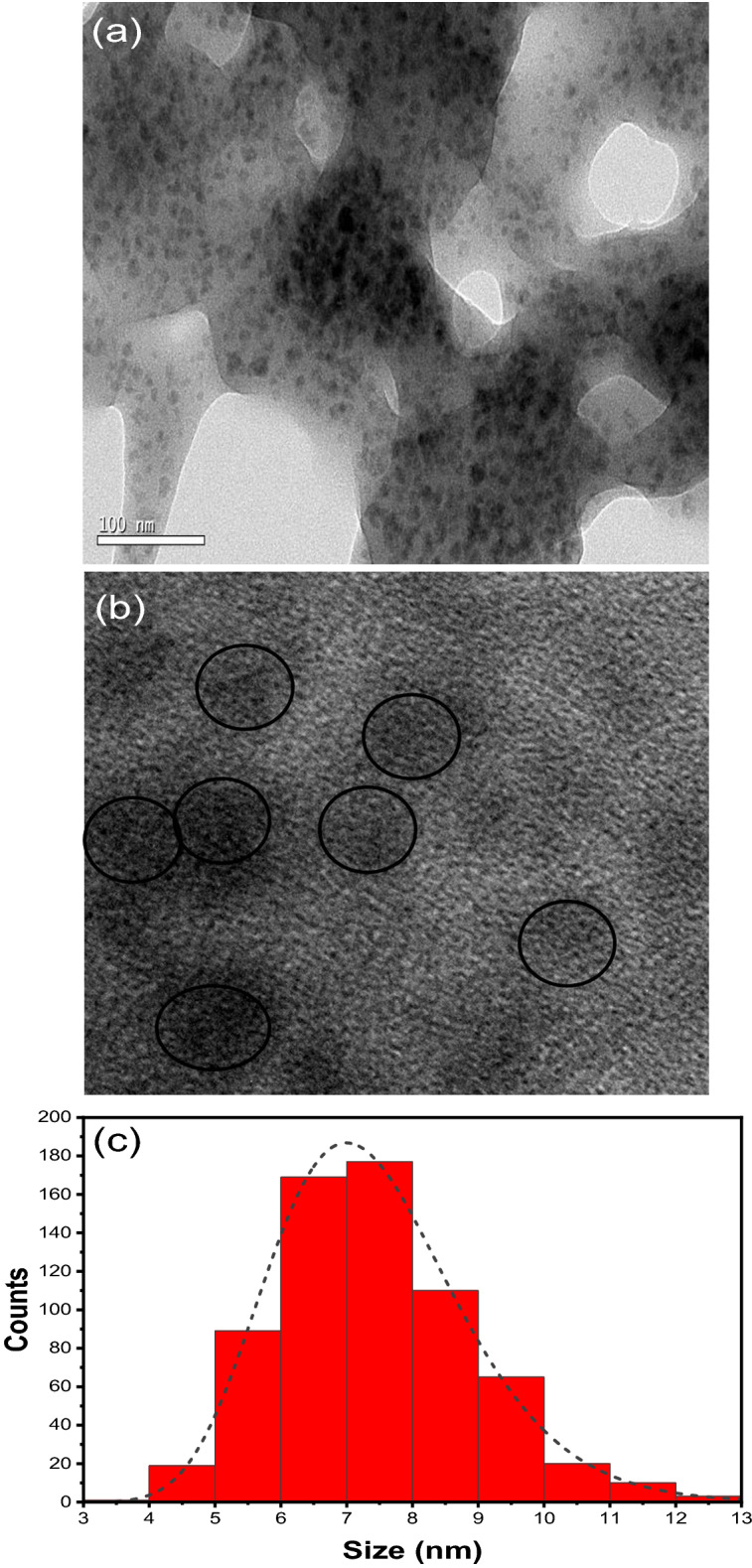
TEM images of CQDs at, (**a**) 100 nm, (**b**) 20 nm resolution, (**c**) size distribution.

HR-TEM analysis was performed to investigate particle morphology and particle size distribution. The TEM image is shown in Fig. 1a. The presence of black spots indicates that CQDs are forming. They are quasi-spherical in shape and have an average size of (7–8) nm (Fig. 1c). The absence of lattice fringes in the HR-TEM image in Fig. 1b indicates that the prepared CQDs are amorphous, which is consistent with previous studies earlier^46,60–63^.

### XPS study

**Figure 2:**
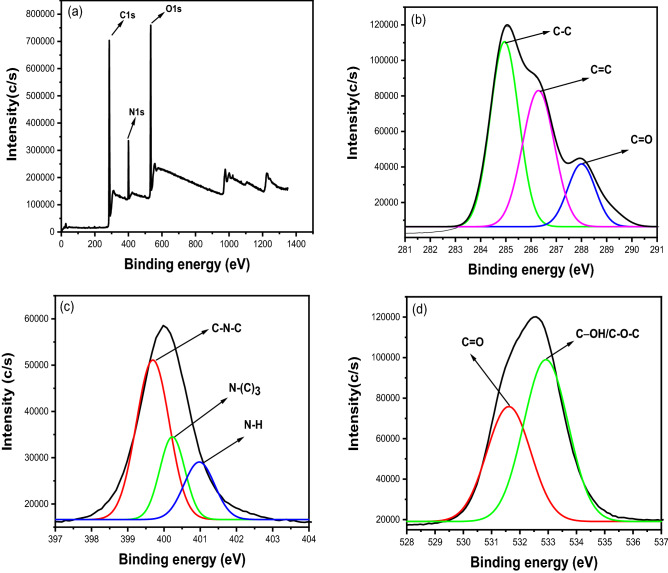
(**a**) Full scan XPS of CQDs. High resolution spectra of (**b**) C 1 s (**c**) N 1 s (**d**) O 1 s.

The elemental composition and surface groups of CQDs were investigated using XPS. Figure 2a shows full scan XPS spectra. The spectrum shows three peaks at 286.12 eV, 400.3 eV, and 532.75 eV which corresponds to C 1 s, N 1 s, and O 1 s respectively^12,46,64^. According to the findings, CQDs are primarily composed of carbon (67.36%), nitrogen (9.91%), and oxygen (22.73%). The high C and O content indicates that the particles have a lot of carboxyl groups on the surface^60^. CQDs have good water solubility due to carboxyl groups and do not require further chemical modification^65^. Oxygen-containing groups may be responsible for their solubility in polar solvents including water^66^. Deconvolution of C 1 s yields three peaks at 284.92 eV, 286.5 eV, and 287.9 eV, corresponding to C–C, C–N/C–O, and C=O in Fig. 2b ^12,46^. In Fig. 2c, the deconvolution of N 1 s produces three peaks at 399.58 eV, 400.2 eV, and 400.99 eV, corresponding to C–N–C, N–(C)3, and N–H, respectively^12,47^. In Fig. 2d, the spectrum of O 1 s shows two peaks at 531.6 eV and 532.9 eV, which are attributed to C=O and C–OH/C–O–C, respectively^46,47^.

FTIR and XPS studies indicate the presence of various functional groups on the surface of these CQDs without any surface modification. However the surface state of CQDs can be altered by the doping of Nitrogen, organic molecules, heteroatom doping e.g. Sulphur, Phosphorous, Boron, Fluorine, etc.^66–72^.

### FTIR studies

**Figure 3:**
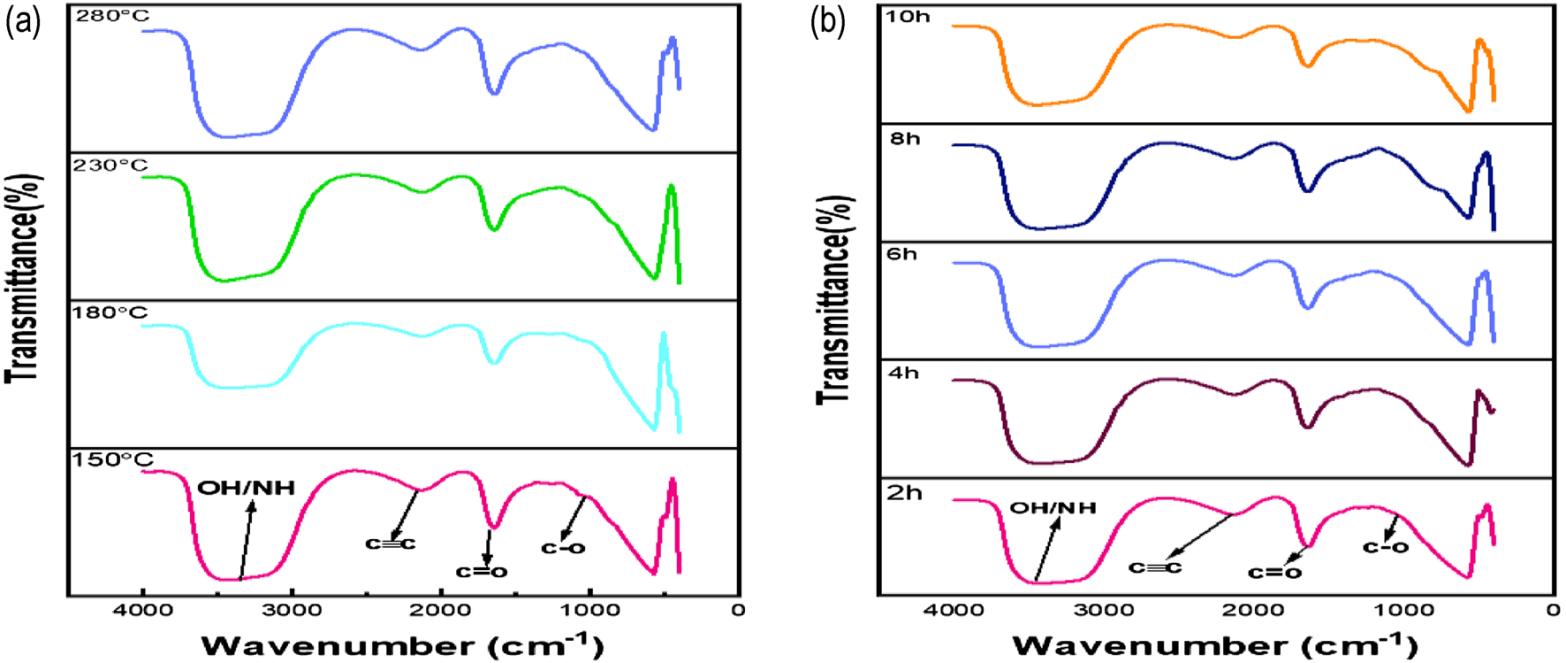
FTIR spectra of CQDs (**a**) at different temperatures, (**b**) at different times.

Figure 3 shows the FTIR spectrum of the prepared CQDS at various hydrothermal temperatures (150, 180, 230, 280 °C) and time (2, 4, 6, 8, 10 h). The absorption band from 3175 to 3551 cm^−1^ is attributed to O–H and N–H stretching vibrations of amine groups^46,65,73^. The peak at 2129 cm^−1^ is assigned to weak C≡C stretching of alkyne. The peak at 1653 cm^−1^ is due to C=O bond stretching^12^. The peak at 1037 cm^−1^ corresponds to C–O stretching^74,75^. FTIR spectrum obtained at different times and temperatures suggests that there is no appreciable change in the spectrum.

### UV–visible and Pl studies

**Figure 4:**
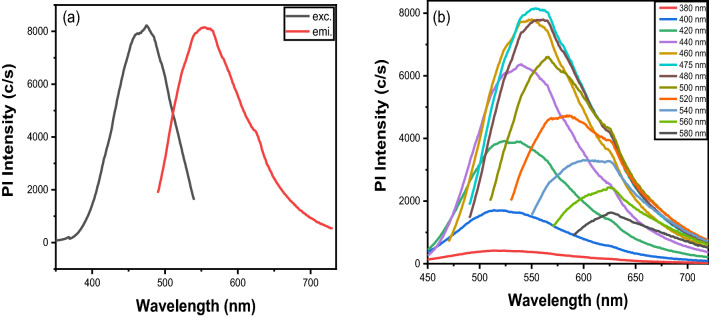
(**a**) Fluorescence excitation (dotted line) and emission spectra (red line) of CQDs. (**b**) Excitation-dependent emission spectra of CQDs.

The UV–visible spectra of CQDs and CQDs with Sn^2+^ ion is displayed in Fig. S1. It demonstrates two shoulder peaks that are located at 275 nm and 330 nm respectively. It's possible that the peak at 275 nm is caused by ח–ח* transitions in the C=C bond, and the peak at 330 nm could be caused by n–ח* transitions in the C=O bond^37^. The Quantum yield of CQDs is calculated using quinone sulfate as a reference^12^. The value comes out to be 38% which is comparable to the studies reported in the literature^76,77^. The fluorescence excitation and emission spectra of CQDs are depicted in Fig. 4a. CQDs exhibit broad and featureless excitation and emission bands rather than characteristic absorption and emission peaks, as shown in Fig. 4a. The excitation wavelength ranges from 380 to 550 nm (in the UV–visible region), with a maximum of 475 nm. And the emission wavelength ranges from 480 to 730 nm, with a maximum of 550 nm. These broad excitation and emission bands could be caused by non-uniform particle sizes and functional groups on the surface of CQDs^60,78,79^. The prepared CQDs exhibit excitation-dependent emission properties. This is depicted in Fig. 4b. We observed emission at various excitation wavelengths across the entire excitation range, from 380 to 580 nm with a gap of 20 nm. There is an increase in PL intensity and a very slight shift of emission peaks to the longer wavelength from 380 to 475 nm. However, the pl intensity decreases from 480 to 580 nm, and emission peaks shift to longer wavelengths. This behaviour could be attributed to the CQDs' varying sizes, random distribution, and the presence of various organic functional groups on their surfaces^44,47,60,65,80–82^. The first reason for excitation-dependent emission is due to non-uniform CQD sizes. Different band gaps correspond to different CQD sizes. When a specific wavelength of light is projected onto CQDs, particles of the same size emit. When other wavelengths are projected, particles of different sizes emit. As a result, the emission is dependent on excitation. The surface state of the CQDs is the second cause. XPS and FTIR studies show that CQD surfaces contain a variety of functional groups.

**Figure 5:**
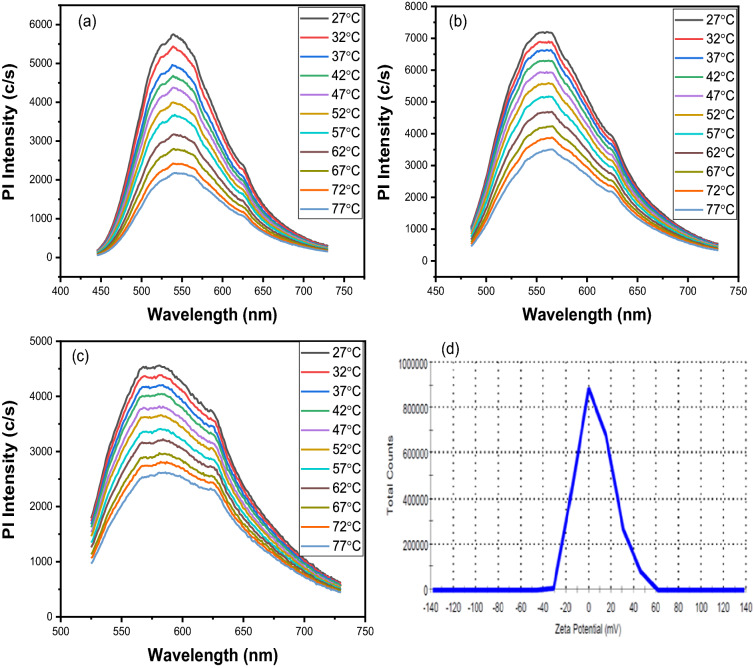
Variation of Pl intensity with the temperature at (**a**) 435 nm, (**b**) 475 nm, (**c**) 515 nm excitation wavelength, and (**d**) Zeta potential (mV).

These functional groups have the ability to generate their own energy levels. As a result, an electron can reach the ground state via photon emission via various routes, resulting in excitation-dependent emission. The intensity of the Pl varies with temperature, ranging from room temperature 27–77 °C with a 5 °C difference. The variation is measured at three different wavelengths: one at maximum excitation (475 nm), one below maximum excitation (435 nm), and one above maximum excitation (515 nm). Figure 5 depicts this. To get a clear picture, they are all chosen at random. The figures show that the intensity of the Pl decreases with increasing temperature, and the variation is consistent across all wavelengths. This decrease in intensity could be attributed to an increase in non-radiative relaxation at high temperatures as a result of thermal activation of non-radiative trapping^83,84^. The zeta potential of the prepared CQDs is shown in Fig. 5d. The obtained zeta potential is 6.57 mV. Its value is in the same order as given in the literature. Its value is close to reported values in the literature in terms of order^85,86^.

**Figure 6:**
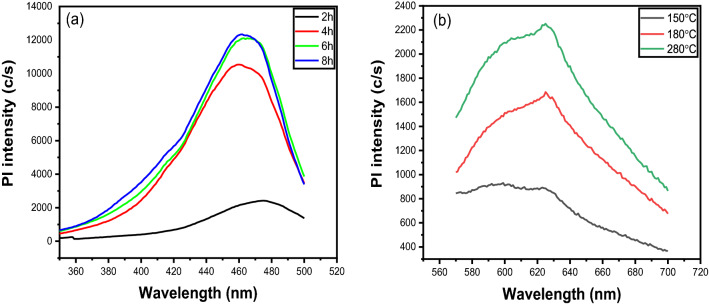
Variation of Pl intensity over (**a**) times, and (**b**) temperature.

Figure 6a,b depicts the change in Pl intensity over synthesis time and temperature. The figure shows that the Pl intensity increases with increasing hydrothermal time and temperature, but there is no shift in the maximum peak position^42,85^. The reason for this could be that as the hydrothermal time and temperature increase, the constituents dehydrate, polymerize, and carbonize, and a greater number of constituents are converted to carbonization^85^.

### Response of CQDs to pH value and solvents

**Figure 7:**
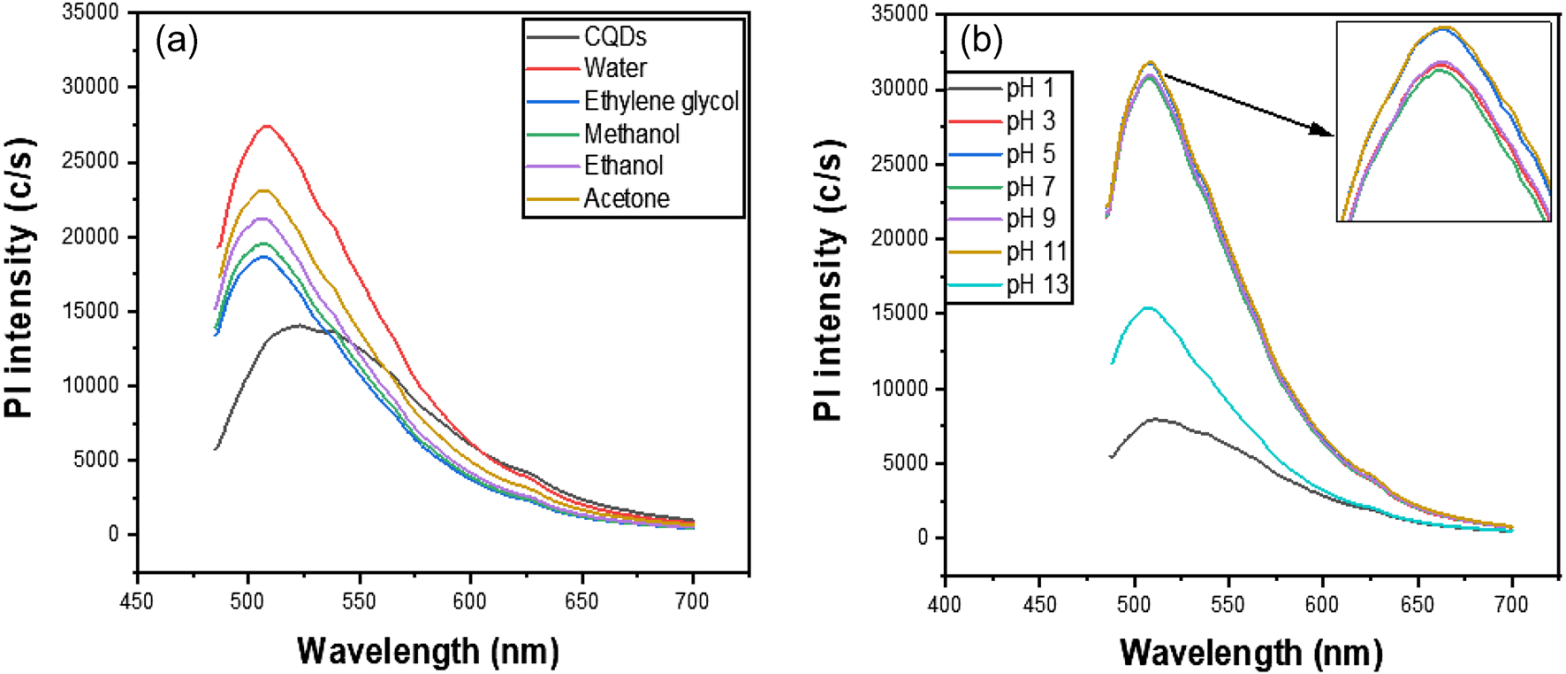
Effect on the Pl intensity of CQDs in (**a**) different solvents and (**b**) different pH environments.

To test the solubility and luminescence properties of CQDs, they were dissolved in various diluted solvents. They were soluble in water, ethylene glycol, methanol, ethanol, and acetone, as well as other polar organic solvents. This could be due to the presence of polar functional groups on the surface of CQDs, such as carboxyl and hydroxyl^87^. Pl intensity is higher in polar solvents than in pure CQDs, and it is greatest in water (Fig. 7a)^85,88,89^. As a result, water is an excellent CQD solvent. The effect of pH on the Pl intensity of CQDs was also investigated. Despite the fact that Pl intensity varies with pH, the maximum peak position was not shifted (Fig. 7b). The Pl intensity decreases significantly in the higher and lower pH regions, but it changes slightly in the pH range of 3–11, indicating that CQDs have good stability in this range and can be explored for potential applications^90^.

### Stability of CQDs

**Figure 8:**
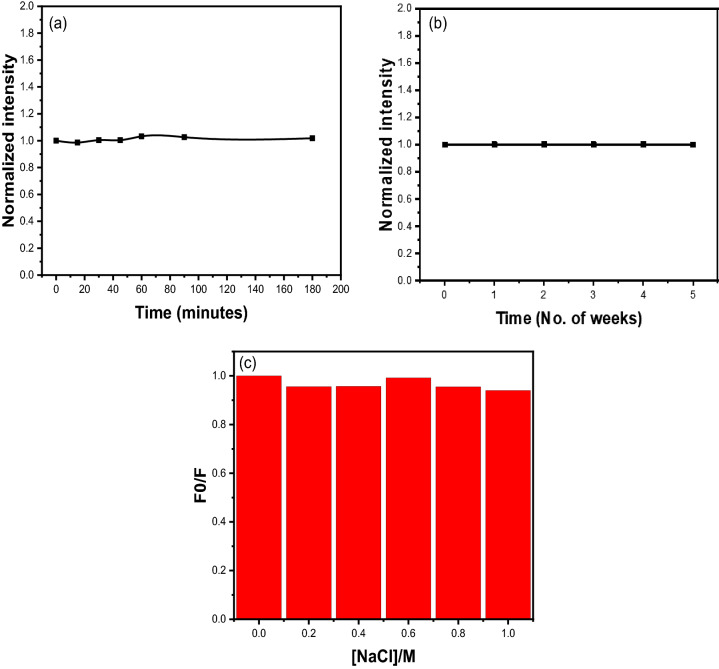
Photostability of CQDs at, (**a**) UV-irradiation, (**b**) storage time, (**c**) in high salt conditions.

CQDs stability is critical for real-world applications. CQD photostability was tested using a variety of methods, including UV irradiation, storage time, and high salt conditions. CQDs were exposed to UV light for 180 min, resulting in a very small change in Pl intensity, as shown in Fig. 8a. They were then stored for 5 weeks, and the Pl intensity was measured at regular intervals; no significant change in the Pl intensity was observed, as shown in Fig. 8b. Similarly, no discernible change in Pl intensity was observed when various concentrations of NaCl (0–1 M) were added (Fig. 8c). In Fig. 8c F0 is the Pl intensity of the blank sample and F is the Pl intensity of CQDs in various NaCl concentrations. All of these findings suggest that CQDs are stable and can be used in practical applications.

### Metal ion detection

**Figure 9:**
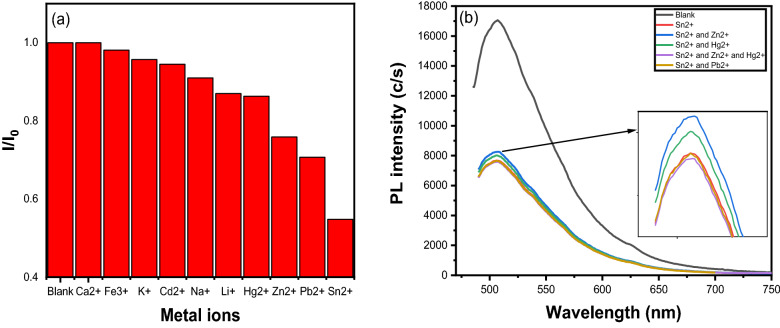
(**a**) Pl intensity of CQDs in different metal ions. (**b**) Comparison of Pl intensity of CQDs in the presence of mixed metal ions.

Selectivity is an important factor in developing an effective sensor for detecting metal ions in aqueous solutions. It is investigated by introducing different metal ions into CQDs, such as Ca^2+^**,** Fe^3+^, K^+^, Cd^2+^, Na^+^, Li^+^, Hg^2+^, Zn^2+^, Pb^2+^, and Sn^2+^ to CQDs. 5 mM metal ions were added to 500µL buffer solution, 100µL CQDs, and the final volume was raised to 2.5 mL by adding deionized water. The Pl spectra were captured at an excitation wavelength of 475 nm. Figure 9a shows the ratio of the intensity of Pl when different metal ions are added to CQDs (I) to the intensity of Pl in a blank sample (I_O_). It was observed that adding Sn^2+^ significantly reduced the intensity ratio. Other metal ions, with the exception of Zn^2+^, Hg^2+^, Pb^2+^, and Li^+^, Pb^2+^ exhibit negligible changes in intensity ratio. This implies that Sn^2+^ has a strong interaction with CQDs. Other metal ions show negligible interference in the detection of Sn^2+^ when mixed with Sn^2+^ (Fig. 9b). As a result, CQDs demonstrated high selectivity for Sn^2+^. Further, the sensitivity toward Sn^2+^ was analyzed by adding different concentrations (0–1 mM) of Sn^2+^ to CQDs and recording the Pl responses at 475 nm excitation wavelength and is shown in Fig. S2. It was observed that Pl intensity decreased with the increased concentration of Sn^2+^ and the maximum peak position did not shift. It indicates that CQDs were sensitive to Sn^2+^. So, the prepared sensor can be used for the detection of Sn^2+^ in the environment. Similar quenching-based sensors have been reported in the literature as well^12,42,87,88,91^. Figure S3 represents the I/I_0_ ratio vs various concentrations of Sn^2+^ ion. It showed a good linear response in the range of 0–50 µM and the calculated limit of detection (LOD) value is 17 µM^92^. The calculations for the LOD are based on the method given in the literature^72^. The whole curve is not linear, which may indicate the presence of static and dynamic quenching in the sensor^12^.

**Scheme 1.**
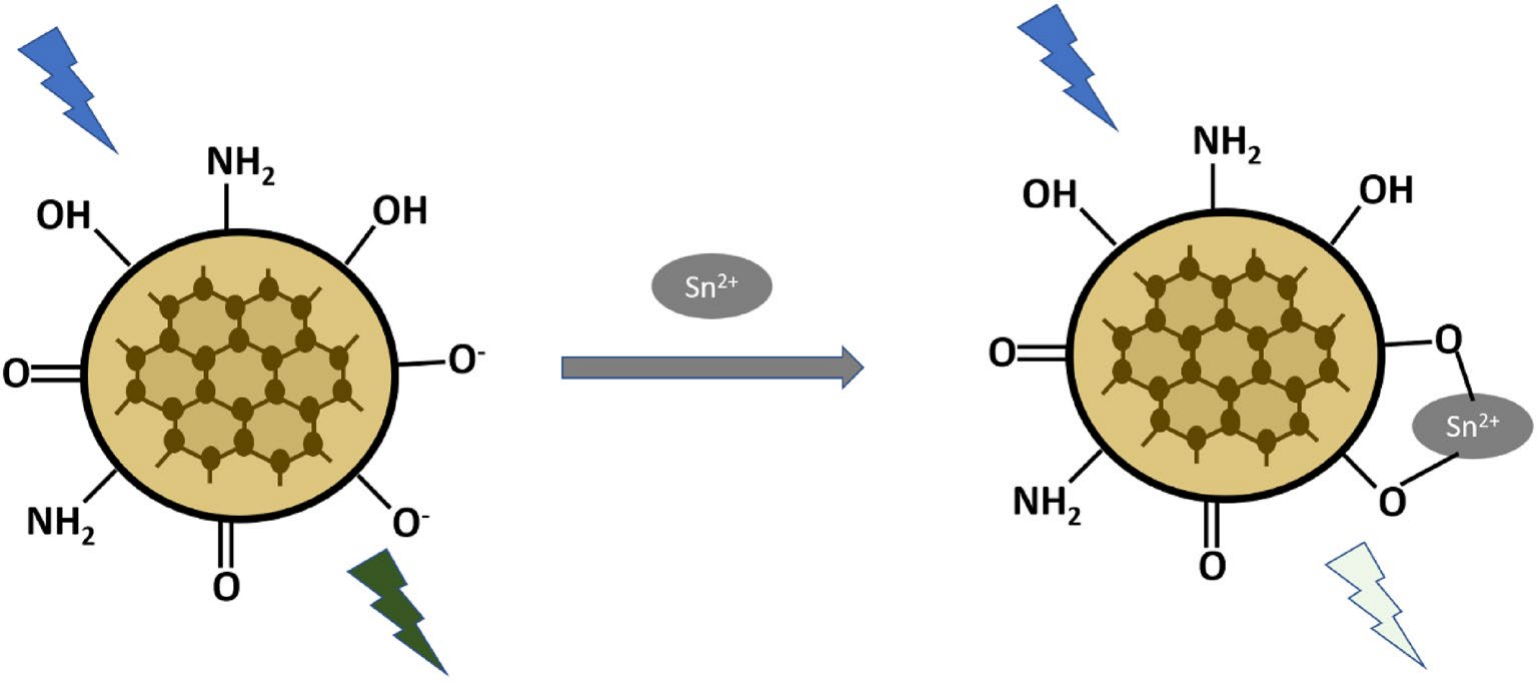
Luminescence quenching mechanism.

The luminescence quenching mechanism is illustrated in Scheme 1.

### Proposed detection mechanism

The proposed quenching mechanism can be elaborated on the basis of UV–Visible and FTIR spectra. UV–Visible spectra are shown in Fig. S1. The peaks at 275 nm and 330 nm correspond to ח–ח* and n–ח* transitions of the C=C and C=O bond respectively depicted in Fig. S4. It was noticed that with the addition of Sn^2+^ the shoulder peaks at 275 nm and 330 nm disappear, which may indicate the interaction of CQDs with Sn^2+^^37^. Furthermore, Comparative studies of FTIR spectrum of CQDs and Sn^2+^ in CQDs is shown in Figs. S5, S6. It reveals the presence of O–Sn–O functional group at 417 cm^−1^ indicating the complex formation between CQDs and Sn^2+^ions^93^. In addition, the quenching of Pl intensity may be due to the presence of various functional groups such as carboxyl, hydroxyl, amine, etc. These groups may enhance metal ion chelation and non-radiative recombination, resulting in intensity quenching^94^.

## Conclusions

We used the hydrothermal method to synthesize CQDs from cow milk in this study. The method is simple and safe for the environment. CQDs prepared in this manner exhibit broad excitation and emission spectra, high quantum yield of 38% excitation-dependent emission, and excellent photostability. The variation in Pl intensity with temperature was also investigated. They were stable when exposed to UV light, when stored for a long time, and when there were high salt conditions. Because of their high sensitivity and selectivity for Sn^2+^, they are used in the detection of Sn^2+^ via the luminescence quenching mechanism. The limit of detection (LOD) value is 17 µM. Because of their excellent properties, they are a promising candidate for detecting Sn^2+^ in the environment.

## Supplementary Information

Supplementary Information 1.

## Data Availability

The datasets generated during and/or analyzed during the current study are available from the corresponding author on reasonable request.
