# Supplementary material for: Synthesis, characterization and potential sensing application of carbon dots synthesized via the hydrothermal treatment of cow milk
Source: Sci Rep. 2022 Dec 28;12:22495. doi: 10.1038/s41598-022-26906-4 (PMC9797560; doi:10.1038/s41598-022-26906-4)
Supplement: Supplementary file 1 — Supplementary Information 1. [file 41598_2022_26906_MOESM1_ESM.docx]

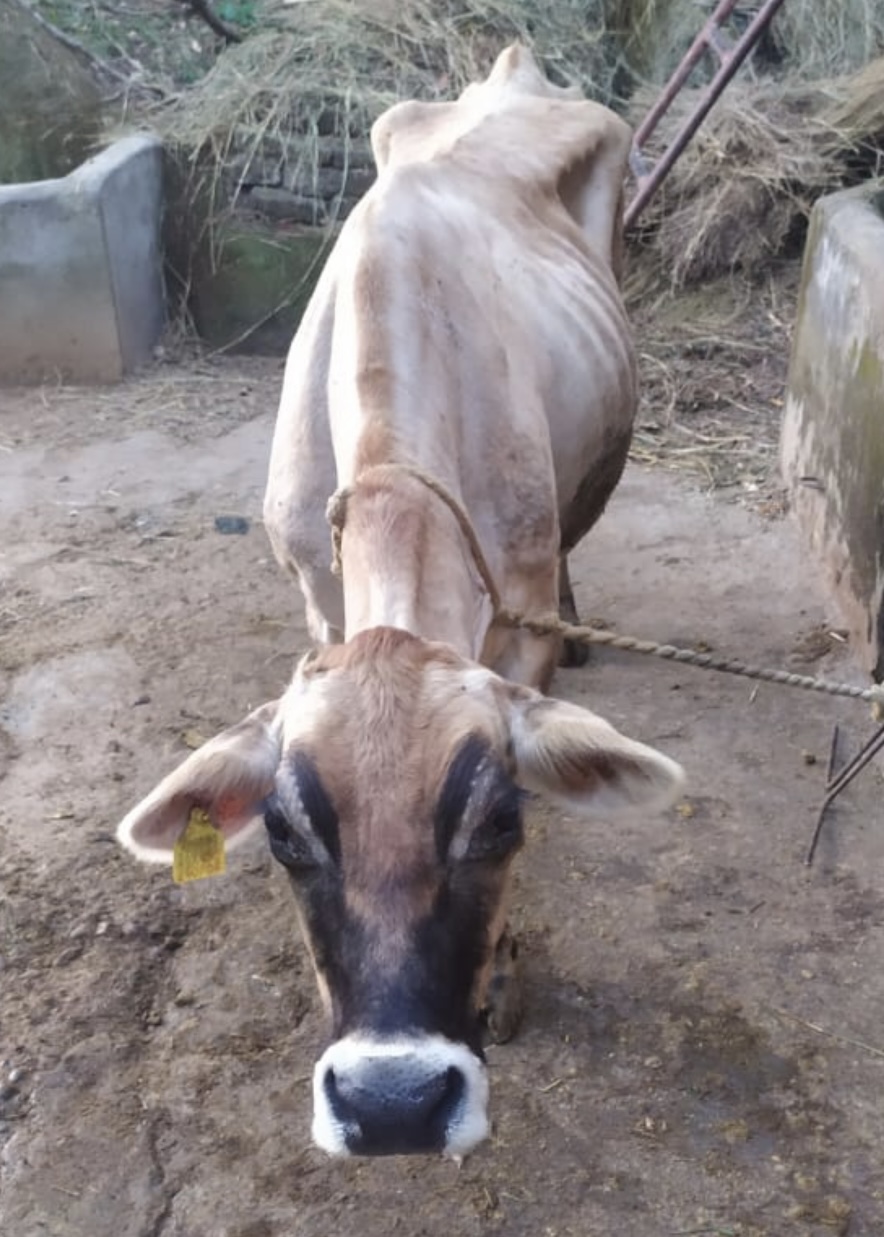


Image. 1

Figure S1. UV-Visible spectrum of CQDs and CQDs + Sn^2+^ ion.

Figure S2. Pl intensity of CQDs in various concentrations of Sn^2+^.

Figure S3. I/I_0_ vs concentrations of Sn^2+^ (I_0_ is the Pl intensity of blank sample and I is the Pl intensity of CQDs in various concentrations of Sn^2+^).


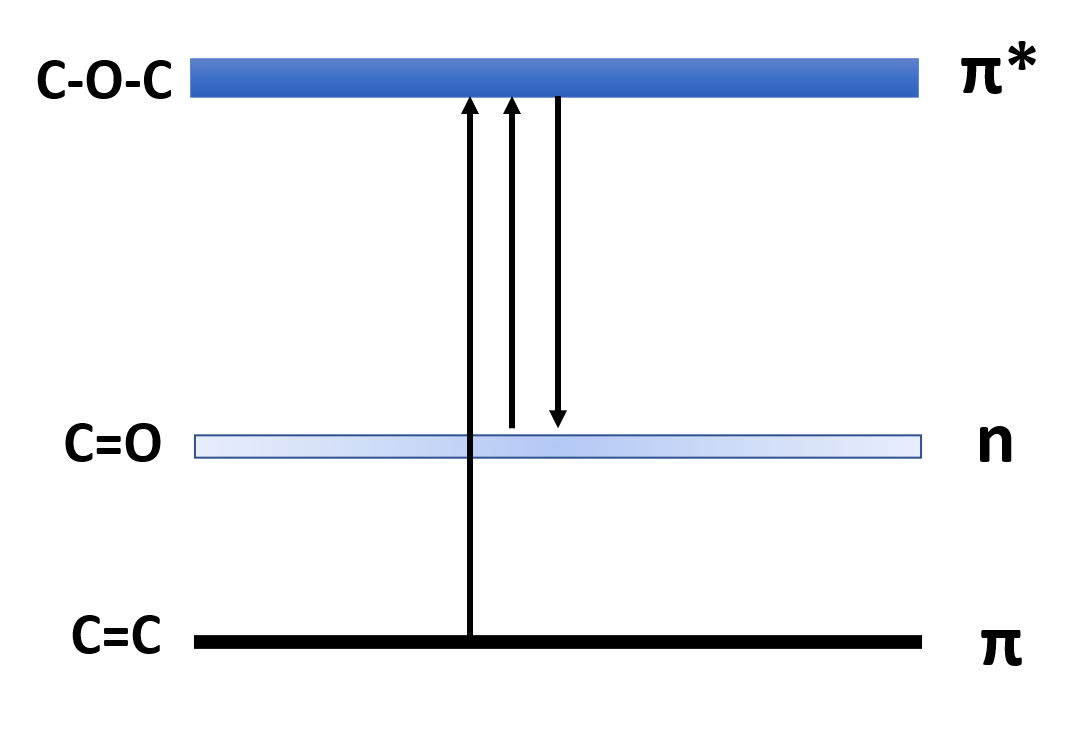


Figure S4. Illustration of π-π* and n-π* transitions.

\

Figure S5. FTIR spectrum of CQDs and CQDs with Sn^2+^ ion.

Figure S6. Zoomed view of FTIR.
